# Green synthesized silver nanoparticles enhance drought tolerance in cotton plants cultured in vitro

**DOI:** 10.1007/s12298-025-01616-z

**Published:** 2025-06-28

**Authors:** Gizem Şafak Baransel, Oğuz Yücel, Eren Yıldırım, Göksenin Kalyon, Serkan Emik, Ayşe Erol, Neslihan Turgut Kara

**Affiliations:** 1 Program of Molecular Biotechnology and Genetics, Institute of Science, Istanbul University, 34116 Istanbul, Turkey; 2 Department of Chemical Engineering, Faculty of Engineering, Istanbul University-Cerrahpaşa, 34320 Avcılar, Istanbul, Turkey; 3 Department of Physics, Faculty of Science, Istanbul University, 34134 Vezneciler, Istanbul, Turkey; 4 Department of Molecular Biology and Genetics, Faculty of Science, Istanbul University, 34134 Istanbul, Turkey

**Keywords:** *Gossypium hirsutum*, Silver nanoparticles, Drought stress, Green synthesis

## Abstract

The study investigated the effects and potential applications of green-synthesized silver nanoparticles (AgNPs) on cotton plants under in vitro drought stress. AgNPs were synthesized using cotton seed oil cake extract (CSOCE) as a stabilizing and reducing agent. The secondary metabolite content of CSOCE was analyzed using High Performance Liquid Chromatography (HPLC). Characterization of synthesized AgNPs was performed using Dynamic Light Scattering (DLS), polydispersity index (PDI), Zeta Potential (ZP), Scanning Electron Microscopy and Energy Dispersive X-Ray Spectroscopy (SEM-EDS), X-Ray Diffraction Analysis (XRD), Ultraviolet-Visible Light Spectroscopy (UV-Vis spectroscopy), and Fourier Transform Infrared Spectrometry (FTIR) analyses. According to SEM, the nanoparticle sizes varied between 50 and 100 nm. ZP was − 28.7 mV and PDI value was 0.65 according to DLS results. The experimental groups were: (1) MS medium (control group), (2) PEG, (3) AgNP, and (4) PEG + AgNP. Plants were transferred to the respective media, cultured for three days, and subsequently analyzed. Morphological parameters including root number, root and shoot lengths, and leaf surface area were measured, while physiological traits such as relative water content, biomass accumulation, osmolyte accumulation, and photosynthetic pigment contents were assessed. Molecular analyses were conducted to examine the relative gene expression of drought stress-associated genes, including *CAT*, *POD*, *Cu/Zn SOD*, *MnSOD*, *MPK17*, *CAX2*, and *IDI-1*. The results demonstrated that the application of AgNPs alleviated the adverse effects of in vitro drought stress on *Gossypium hirsutum* plants. These findings suggest that green synthesized AgNPs hold significant potential as agents to mitigate drought stress in plants.

**Supplementary Information:**

The online version contains supplementary material available at 10.1007/s12298-025-01616-z.

## Introduction

Nanotechnology typically involves materials sized 1–100 nm, the nanoscale. A key concept is the emergence of new properties as material size decreases, which alters material characteristics and broadens their applications (Mansoori 2005).

Nanoparticle synthesis methods are broadly classified into bottom-up and top-down approaches (Baig et al. 2021). Green synthesis utilizing plant extracts stands out among biological methods as a cost-efficient, fast, and scalable strategy, owing to the presence of bioactive phytochemicals. (Iravani 2011; Fahim et al. 2024). Plant extracts are preferred reducing agents due to the presence of effective phytochemicals such as ketones, flavonoids, amides, carboxylic acids, aldehydes, terpenoids and phenols could reduce metal salts to metal nanoparticles (Can 2020; Rajabi et al. 2020). The use of plant extracts in green synthesis has gathered increasing interest as simple, efficient, cost-effective, and convenient method as well as an alternative to chemical synthesis methods for nanoparticle production (Aguilar 2012).

Gossypium, a member of the Malvaceae family, consists of 52 species, including 46 diploid species grouped into eight genome groups (A, B, C, D, E, F, G, and K) and six tetraploid species with the AD genome (Page et al. 2013). Allotetraploid *Gossypium hirsutum* L., being the most widely cultivated fiber crop of high commercial value, is adaptable to distinct climatic conditions. The increasing global demand for cotton and its products, driven by its extensive application in the textile industry and edible oil production, underscores its economic significance (Campbell et al. 2010).

Stress in plants describes conditions that negatively affect the growth, development, or productivity of plants. Complex plant responses are being triggered under stress. Including altered gene expression, cellular processes, changes in growth rates and crop yield. Stress in plants can be sub-titled into two as biotic and abiotic stresses, considering the factor causing the stress (Lichtenthaler 1996). Drought stress in plants develops because of water deficiency. Water deficiency is the situation where the plant’s water needs cannot be met through sufficient rainfall or irrigation. It is possible to divide the plant’s responses to drought stress into three groups according to their basic mechanisms: molecular, biochemical and physiological responses (Oguz et al. 2022). As a result of the increased frequency and intensity of abiotic stresses resulting from climate change, the development and production of most plants have been affected. Prolonged and severe drought conditions in numerous cotton-growing regions have made drought the most significant challenge to global cotton production. Since cotton is a product of tropical and subtropical climates, which are regions more prone to drought stress, its production is under threat of long drought periods predicted in the future (Ul-Allah et al. 2021).

The ability of use of AgNPs to increase the tolerance of plants to various stresses has been evaluated by researchers. The effects of AgNPs on biotic stress caused by *Meloidogyne javanica* in tomato plants were investigated through a comparative analysis of different synthesis methods (Kordsholie et al. 2025). Several studies have demonstrated the positive effects of silver nanoparticles (AgNPs) on plant growth. Iqbal et al. (2019) reported that AgNPs synthesized using *Moringa oleifera* extract enhanced wheat growth under heat stress, with 50 and 75 mg/L concentrations being particularly effective. Abasi et al. (2022) found that AgNPs significantly improved germination rates and salt tolerance in *Cuminum cyminum* L. seeds under salinity stress. Similarly, Almutairi (2016) showed increased resistance in tomato seeds exposed to NaCl stress, leading to higher germination rates, fresh and dry biomass, and root length. Sharon et al. (2010) highlighted AgNPs’ role in repelling soil and hydroponic microorganisms, enhancing plant growth, and protecting against diseases through foliar application. While AgNPs have been shown to mitigate salt stress (Almutairi 2016; Abou-Zeid and Ismail 2018), their effects under drought stress remain less explored. Hojjat (2016) reported that AgNPs improved water balance, shoot growth, biomass, and germination in lentils under drought conditions.

With this study, it was aimed to synthesize AgNPs using the green synthesis method using cottonseed oil cake (CSOC), which is a waste of the cotton industry, and to investigate the potential effects of these synthesized AgNPs in mitigating drought stress on cotton plants. The fact that the material used in nanoparticle synthesis is of waste origin offers a perspective that supports sustainability. Drought conditions, which are increasing especially because of global warming, are one of the biggest problems today. Studies addressing AgNP effects on plant stress conditions are quite limited. Cotton plant is a plant that grows in humid environments and losses occur in different periods as a result of drought stress. This study investigates the morphological, physiological, and gene expression level effects of AgNPs derived from cotton plant waste on the adverse impacts of drought stress when applied to cotton plants.

## Materials and methods

### Preparation of cotton seed oil cake extract and determination of its secondary metabolite content

CSOC was used as a reducing agent for the green synthesis of AgNPs. CSOC, purchased from Çiçek Kardeşler Food Industry and Trade Ltd., was sourced from İzmir Bağ Yağları Industry and Trade Ltd. and harvested during the August-October 2023 term.

CSOCE was prepared with distilled water at a concentration of 10% (w/v) by modifying the method used by Alfuraydi et al. (2019), 90 g of distilled water was added to 10 g of CSOC and kept in a shaker at 120 rpm for 24 h. At the end of the incubation period, the mixture was filtered using filter paper followed by a 0.22 µm membrane filter. The prepared CSOCEs were stored at 4 °C to be used in the green synthesis reaction.

HPLC analysis was performed to investigate the chemical content of 10% CSOCE. A total of 10 standard substances were used in the analysis, including protocatechuic acid, gentisic acid, p-coumaric acid, polydatin, coumarin, routine, aloe-emodin, rhein, chrysophanol and physcion. HPLC analysis was performed with the HPLC device (Shimadzu^®^). The HPLC system used includes a pump (LC-10AD VP), column oven (CTO-10AS), degassing unit (DGU-20 A), diode array detector (SPD-M20A), and autosampler (SIL-20 A).

### Green synthesis of AgNPs

For green synthesis, the method used by Govarthanan et al. (2016) was applied with modifications. Five mM AgNO_3_ ( MKCN7884, Sigma-Aldrich) solution was prepared, and the pH value was adjusted between 5.7 and 5.9. Four ml 10% (w/v) CSOCE was added to 96 ml 5 mM AgNO_3_ solution. Water was used instead of CSOCE for control conditions. The resulting mixtures were incubated in a shaking incubator at 180 rpm, 26 °C for 8 h. Samples were centrifugated at 12000 *x*g for 20 min.

### Dynamic light scattering (DLS) and zeta potential analysis (ZP)

ZP, PDI and particle size of the synthesized AgNPs were evaluated using DLS analysis. The measurements were performed with a particle size and zeta potential analyzer (Malvern Instruments, Zetasizer Nano ZS90, UK). For analysis, samples were prepared by dissolving the nanoparticles in dH₂O at a concentration of 2 mg/mL. All measurements were taken at 25 °C with a scattering angle of 90°.

### Scanning electron microscopy and energy dispersive x-ray spectroscopy (SEM-EDS)

The sample was washed 3 times with acetone (50% solution) (Merck, 1.00013.2500). The size and shape of AgNPs were determined by SEM (FEI, Versa 3D™ DualBeam™) equipped with a EDS detector.

### X-ray diffraction analysis (XRD)

The crystal structure of the AgNPs was determined using XRD (Polycrystalline XRD, T&T TT-90), with Cu Kα radiation (λ = 1.54060 Å) in the 2θ range of 4 − 90° with a step size of 0.01º.

### Ultraviolet-visible light spectroscopy (UV-Vis)

A spectrophotometer with microplate reader (BioTek™ Eon Microplate Spectrophotometer) was used for spectroscopic characterization of AgNPs. UV-VIS was carried out with nanoparticles in colloidal form in solution after an 8-hour incubation period of green synthesis. Samples (200 µl) taken from the conical flasks were placed into the plate wells. Reading was performed in the wavelength range of 300–700 nm using step size of 2 nm and dH_2_O was used as a blank.

### Fourier transform infrared spectrometry (FTIR)

In terms of characterization the chemical structure of AgNPs FTIR analysis was performed using a FTIR spectrometer (JASCO, FT/IR 4700) with range of 400–4000 cm⁻¹.

### Plant growing conditions

*Gossypium hirsutum*, registered variety Harem 2 seeds, obtained from the Ministry of Agriculture and Forestry, Cotton Research Institute Directorate (Nazilli), were used. Plant seeds were germinated on Murashige and Skoog (MS) medium (Murashige and Skoog 1962). Media called PEG, AgNP and PEG + AgNP were used in experimental conditions. MS medium without NPs and drought treatment was used as a control. For the control group, MES and DMSO were added to the MS medium to ensure the same condition as all other conditions. PEG and PEG + AgNP media were prepared in two layers, with a PEG 6000 concentration of 15% w/v, according to the method used by Van der Weele et al. (2000), AgNP was added to AgNP and PEG + AgNP media by dissolving it in DMSO to a final concentration of 50 mg/L.

Cotton seeds were sterilized (Aydın and Turgut Kara 2025) and germinated on MS medium in a plant growth cabinet [24 °C ± 2, 16 h light/8 hours dark, 1400 lux light intensity]. Cotton plants germinated for 4 days in a plant growth cabinet then were transferred to MS, PEG, AgNP and PEG + AgNP media. Plants transferred to the relevant media were kept in the same climate chamber conditions for 3 days.

### Morphological analysis

The root and shoot lengths, number of roots, and leaf surface areas of the plants were analyzed. The number of roots was counted for each plant. Root and shoot lengths were measured directly using a ruler. The leaf surface area was calculated using the formula for cotton plants (Su et al. 2015).

### Physiological analysis

Relative water content (RWC), biomass accumulation, osmolyte accumulation, chlorophyll and carotenoid contents were determined. RWC was calculated following by the method Smart and Bingham has been explained (1974).

Fresh weight (FW) biomass, and dry weight (DW) biomass were dried weighed to determine dry weights samples dried 48 h on 70 °C (Gupta et al. 2018).

Osmolyte accumulation has been analysied 25 mg of tissue consisting of leaves was transferred to centrifuge tubes. The samples were kept at -20 °C overnight. Leaf extract was obtained by centrifugation at 4 °C for 30 min. 15 µl of plant sap and 135 µl of dH_2_O were loaded into the probe of the “semi-micro” osmometer (Knauer, K7400) device, and the osmolarity of the sample was expressed as mOsmol/kg (Uçarlı and Gürel 2020).

For measuring the concentrations of chlorophyll a (Chl *a*), chlorophyll b (Chl *b*), total chlorophyll content (Chl *a + b*), and carotenoid pigments, leaf samples (100 mg) were homogenized in 10 ml of chilled 80% acetone using a pre-chilled mortar and pestle. Tissue debris was removed by centrifugation at 8000 *xg* for 10 min at 4 °C. The absorbance of 200 µl of the supernatants was measured using a spectrophotometer with a microplate reader (BioTek™ Eon Microplate Spectrophotometer) at 470 nm (OD470), 646.8 nm (OD646.8), and 663.2 nm (OD663.2). An 80% acetone solution was used as the reference. The concentrations were calculated in mg per gram of fresh weight (FW) using the formulas reported by Gupta et al. (2018).

### Total RNA isolation and cDNA synthesis

For each condition, 100 mg of sample was ground in liquid nitrogen using a pre-cooled mortar and pestle. RNA isolation was performed using home-made Trizol reagent, following the protocol described by Valach (2016). DNase I treatment was applied to eliminate DNA contamination. Finally, NaAc re-precipitation was used to remove remaining impurities (Manickavelu et al. 2007).

cDNA synthesis was performed from the isolated RNAs using the High-Capacity cDNA Reverse Transcription kit (Thermo Scientific, 4368814). The synthesis was performed on a PCR device (BIO-RAD, T100 Thermal Cycler) following the recommended manufacturer’s protocol.

### Real-time polymerase chain reaction and gene expression analysis

Real-time polymerase chain reaction (qPCR) was utilized for comparative analysis of gene expression. The primers used for the selected genes are listed in Table 1, and *UBQ7* used as the reference gene (Wang et al. 2013). The PCR protocol was conducted as follows: an initial hold was performed at 50 °C for 1 min, followed by a denaturation step at 94 °C for 30 s. The annealing step was performed at either 50.5–57 °C, depending on the primer specificity, for 30 s. The extension phase was conducted at 72 °C (40 cycles). The protocol concluded with a final hold at 40 °C for 30 s. The reaction was executed on the CFX96 Touch Real-Time PCR System (BioRad).  Ct values ​​ from the qPCR results were calculated with the 2^−∆∆Ct^ method (Schmittgen and Livak 2008) and relative gene expression coefficients were obtained referring to the *UBQ7* gene.

**Table 1:** The primer sequences used in gene expression analysis

Primers		Sequence	Annealing Temperature	Product Size (bp)
*CAT*	Forward Reverse	5′-GATCCCTACAAGCACCGTCC-3′ 5′-GGAATCCGCTCCCTGTCAAA-3′	57 °C	176
*POD*	Forward Reverse	5′-GTGTTGTCTACGGTTGAGTTAG-3′ 5′-GAAGAAGTGTCGTCTAGGAGTA-3′	57 °C	140
*MnSOD*	Forward Reverse	5′-CACGTCAACCACTCCATTT-3′ 5′-CACCCTCAGCATTCATCTTC-3′	50.5 °C	145
*Cu/ZnSOD*	Forward Reverse	5′-TCGGAAGTTGAAGGCGTTGT-3′ 5′-TGCTCCTGTAGACATGCACC-3′	50.5 °C	150
*MPK17*	Forward Reverse	5′-CATTTTACTCCAACTACACCCC-3′ 5′-TCCACCACATTCTTCCCTG-3′	57 °C	106
*IDI-1*	Forward Reverse	5′-TGAACCGTGACCAACTGAAGGAGT-3′ 5′-TTCAGGGTCCCTTTCTCGACATGA-3′	50.5 °C	139
*CAX3*	Forward Reverse	5′-CGTCATCTCTTTGCTCTCTG-3′ 5′-GATCCTAATGCCACACCTAAA-3′	57 °C	189
*UBQ7*	Forward Reverse	5′-AAGCCCAAGAAGATCAAGCA-3′ 5′-CGCATTAGGGCACTCTTTTC-3′	50.5 °C, 57 °C	114

### Statistical analyses

The data obtained from the morphological, physiological and molecular analysis were statistically analyzed and graphed using the GraphPad Prism^®^ 9.0 program One Way ANOVA (“One Way AVOVA”) analysis. Turkey’s multiple comparisons test results with adjusted *p* < 0.05 were considered significant.

## Results

### HPLC analysis of cotton seed oil cake extract (CSOCE)

Ten standard substances were utilized in the analysis: protocatechuic acid, gentisic acid, p-coumaric acid, polydatin, coumarin, rutin, aloe-emodin, rhein, chrysophanol, and physicon. The analysis results revealed that protocatechuic acid and coumarin were not detected in CSOCE, while rhein was identified as the secondary metabolite with the highest content (Table 2).

**Table 2:** CSOCE content determined by HPLC analysis are given the average amounts for each secondary metabolite with standard errors (SE)

Secondary Metabolite Type	Average Amount (µg/mg) ± SE
Protocatechuic Acid	Not detected
Gentisic Acid	0.005 ± 0.000
p-Coumaric Acid	0.024 ± 0.001
Polydatin	0.014 ± 0.000
Coumarin	Not detected
Rutin	0.011 ± 0.003
Aloe-emodin	0.021 ± 0.002
Rhein	0.215 ± 0.002
Chrysophanol	0.184 ± 0.018
Physcion	0.182 ± 0.019

### Green synthesis of silver nanoparticles

**Fig. 1:**
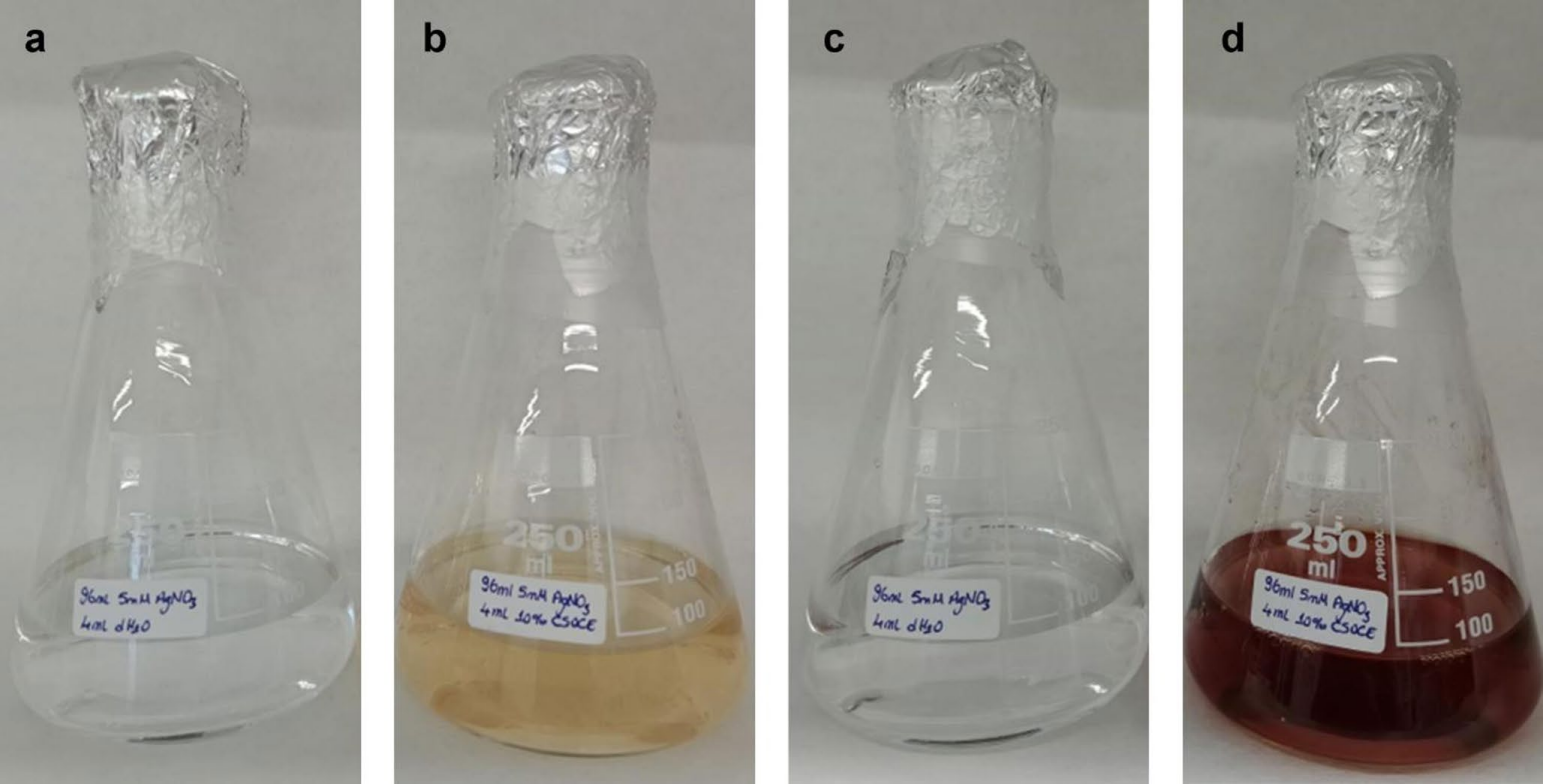
Color change in green synthesis because of reduction and indicating SPR **a** Control (AgNO_3_ + dH_2_O) before the incubation of green synthesis; **b** Sample (AgNO_3_ + CSOCE) before the incubation of green synthesis; **c** Control (AgNO_3_ + dH_2_O) after the incubation of green synthesis; **d** Sample (AgNO_3_ + CSOCE) after the incubation of green synthesis

In green synthesis, color change is considered an indicator that silver ions are reduced to AgNPs by the effect of Surface Plasmon Resonance (SPR) (Arokiyaraj et al. 2015). Color change in the green synthesis reaction before and after incubation was observed as a color transformation from yellow to brown (Fig. 1).

### Characterization by UV–Vis spectrophotometer

**Fig. 2:**
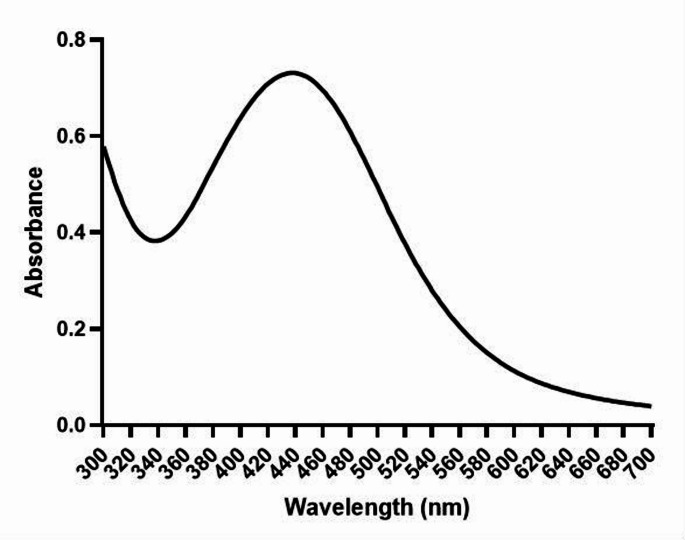
UV-VIS absorption spectrum of the green synthesized AgNPs, a sharp SPR on 430–450 nm has been observed

The formation of a brown color can be attributed to the SPR effect and the reduction of Ag^+^ ions to Ag⁰ by the aqueous extracts. A sharp SPR band was observed at approximately 430–450 nm (Fig. 2), which is specific to AgNPs (Ramalingam et al. 2016). Based on the presence, shape, and position of a single peak, demonstrated the synthesized nanoparticles are spherical. This result is further supported by the SEM image of the sample.

### Characterization by DLS, PDI value and zeta potential

**Fig. 3:**
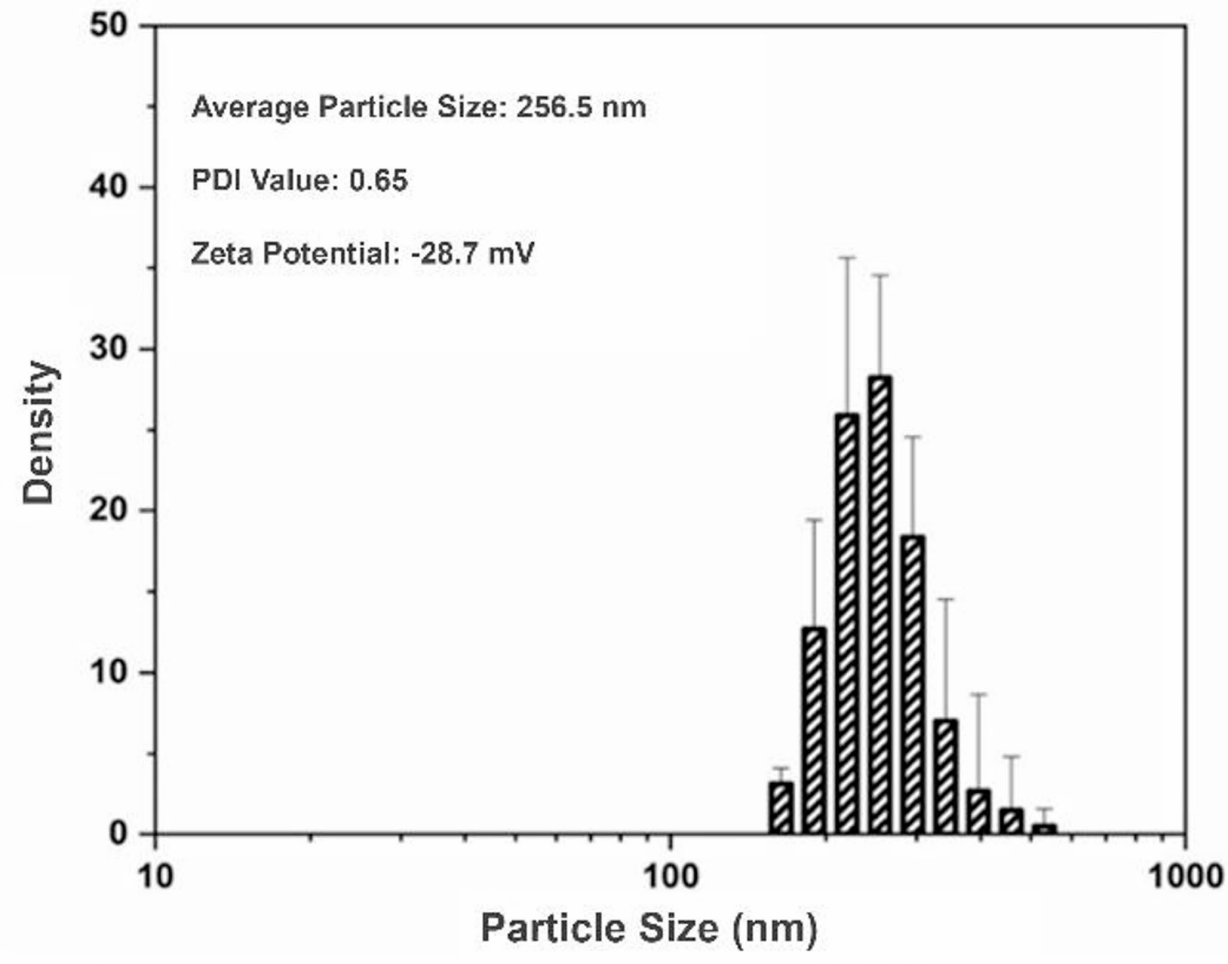
DLS measurements of green synthesized AgNPs, have 256.5 nm average size, the PDI value is 0.65, and the ZP is-28.7 mV

As a result of DLS analysis, the average particle size was determined to be 256.5 nm, the PDI value was 0.65, and the ZP was measured as -28.7 mV (Fig. 3).

### Characterization by SEM and EDS

**Fig. 4:**
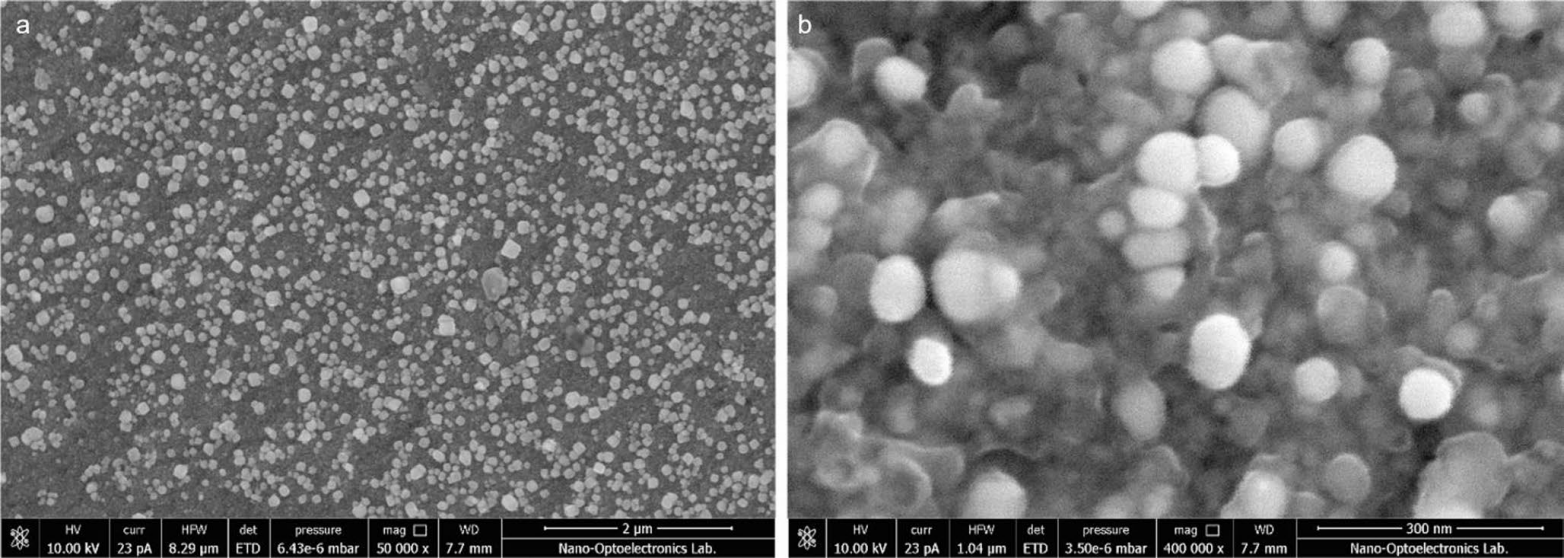
SEM image of green synthesized AgNPs. **a** In 2 μm scale SEM image; **b** In 2 μm scale SEM image

**Fig. 5:**
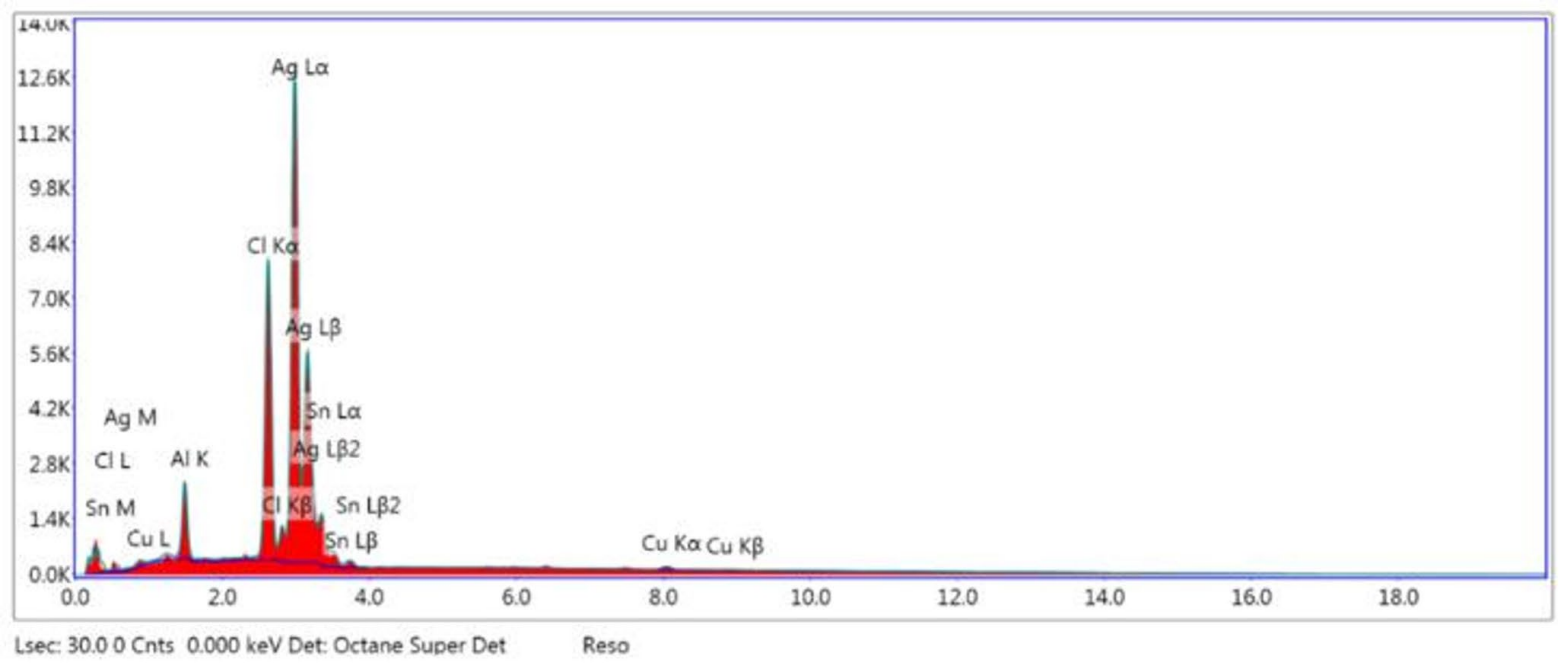
EDS analysis of green synthesized AgNPs, kV: 30 Mag: 60,000 Takeoff: 32 Live Time(s): 30 Amp Time(µs): 7.68 Resolution: (eV) 128.6

SEM analysis revealed that the particles exhibited a spherical morphology, with nanoparticle sizes ranging between 50 and 100 nm. Elemental composition analysis of the SEM-recorded images was conducted using EDS (Figs. 4 and 5). The results indicated the presence of silver, identified by the L characteristic peak, which appeared at 3 kV. The mass fraction of silver was calculated to be approximately 64.44%.

### Characterization by XRD

**Fig. 6:**
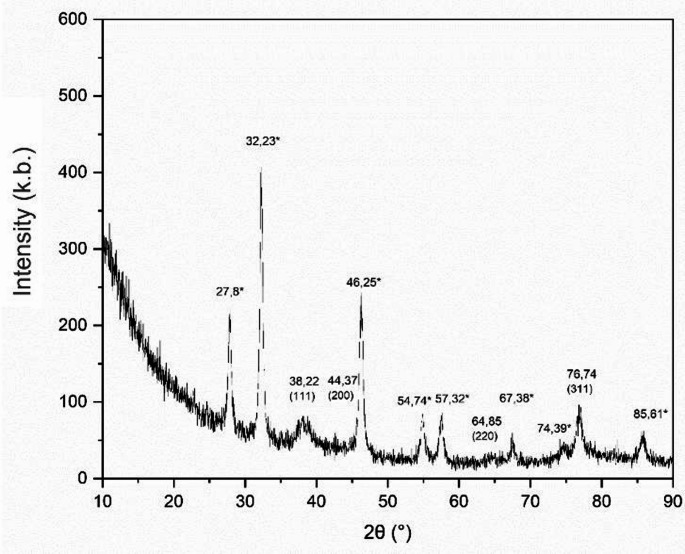
XRD analysis of green-synthesized AgNPs showed diffraction peaks corresponding to Bragg reflections (111), (200), (220), and (311), which belong to the face-centered cubic (fcc) structure of Ag

The XRD pattern of AgNPs showed diffraction peaks at 2θ = 38.22°, 44.37°, 64.85°, and 76.74°, corresponding to the (111), (200), (220), and (311) Bragg reflections, indicating a face-centered cubic (fcc) structure of Ag (JCPDS file number 04-0783) (Fig. 6). The additional peaks marked with an asterisk in Fig. 6 are attributed to the formation of AgCl.

### Characterization by FTIR

**Fig. 7:**
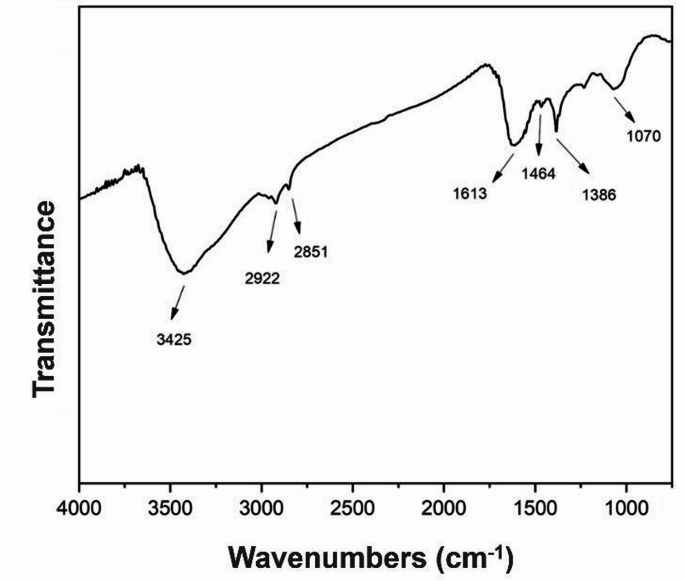
3425 cm^− 1^, 2922 cm^− 1^, 2851 cm^− 1^, 161, 1464 cm^− 1^, 1386 cm^− 1^, and 1070 cm^− 1^ were observed on FTIR spectrum of green synthesized AgNPs

Peaks at 3425 cm^− 1^, 2922 cm^− 1^, 2851 cm^− 1^, 161, 1464 cm^− 1^, 1386 cm^− 1^, and 1070 cm^− 1^ detected by FTIR analysis. Peaks representing various chemical bonds were determined (Fig. 7).

### Morphological effects of AgNPs under drought stress

**Fig. 8:**
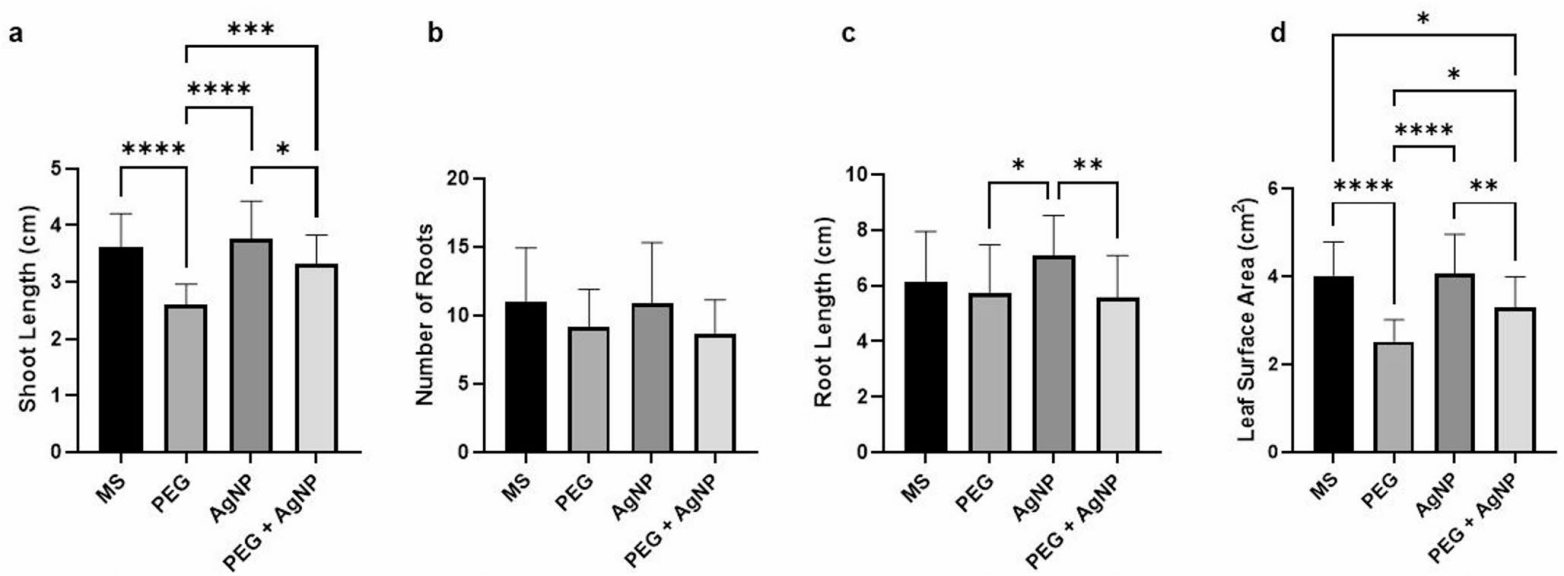
Results of morphological analysis. (**a** Shoot lengths of the plants, **b** Number of roots of the plants, **c** Root lengths of the plants, **d** Leaf surface areas of the plants). Asterisks represent statistically significant variations (*p ≤ 0.0001, **p ≤ 0.001, **p ≤ 0.01, *p ≤ 0.05), whereas ns (not significant), which is not shown in the figures

No significant difference was observed in root numbers among the four tested conditions. However, plants treated with AgNPs exhibited significantly longer root lengths compared to those under the PEG and PEG + AgNP treatments. In the PEG condition, shoot length decreased significantly compared to the MS condition. Notably, the PEG + AgNP treatment resulted in a significant increase in shoot length compared to the PEG condition. AgNPs alone did not lead to a significant difference in leaf surface area (LSA) compared to the MS condition. Under PEG treatment, a significant reduction in LSA was observed due to drought stress. However, the PEG + AgNP application significantly improved LSA compared to the PEG condition, although it remained lower than the LSA values recorded in the MS and AgNP treatments (Fig. 8). The mean values for all morphological parameters, along with their respective standard deviations, are presented in Table 3.

**Table 3:** Mean values and standard errors (± SE) of morphological parameters measured under MS, PEG, agnp, and PEG + AgNP treatments

Morphological Analysis	MS	PEG	AgNP	PEG + AgNP
Number of Roots	11.04 ± 0.7756	9.208 ± 0.5483	10.92 ± 0.8592	8.667 ± 0.5096
Root Length (cm)	6.152 ± 0.3612	5.754 ± 0.3501	7.096 ± 0.2813	5.592 ± 0.3037
Shoot Length (cm)	3.616 ± 0.1169	2.608 ± 0.0719	3.769 ± 0.1288	3.317 ± 0.1052
Leaf Surface Area (cm^2^)	4.019 ± 0.1752	2.522 ± 0.1169	4.071 ± 0.1994	3.297 ± 1.631

### Physiological effects of AgNPs under drought stress

A significant decrease in RWC was observed in plants subjected to PEG application due to drought stress. PEG + AgNP application significantly increased the RWC compared to PEG. Osmolyte accumulation was highest in plants under PEG conditions, while plants treated with AgNPs exhibited the lowest levels of osmolyte accumulation. PEG + AgNP resulted in a significant reduction in osmolyte accumulation compared to PEG.

Drought stress under PEG conditions caused a reduction in chlorophyll a, chlorophyll b, and total chlorophyll levels. For all three chlorophyll parameters, PEG + AgNP application significantly increased chlorophyll content compared to PEG alone, effectively mitigating the drought-induced reduction in chlorophyll. In terms of carotenoid content, differences were determined between PEG and both AgNP and PEG + AgNP conditions, but no significant differences were found between PEG and MS conditions. Similarly, no significant differences were detected in chlorophyll and carotenoid contents between AgNP and PEG + AgNP treatments.

The FW of plant shoots was decreased under PEG conditions compared to all other conditions. No significant difference in shoot FWs was observed between MS and AgNP. However, PEG + AgNP plants exhibited significantly higher shoot FW compared to both PEG and MS or AgNP. Despite the significant reduction in shoot FW under drought stress in PEG plants, the addition of AgNPs under drought conditions led to the development of plants with higher shoot FWs.

**Fig. 9:**
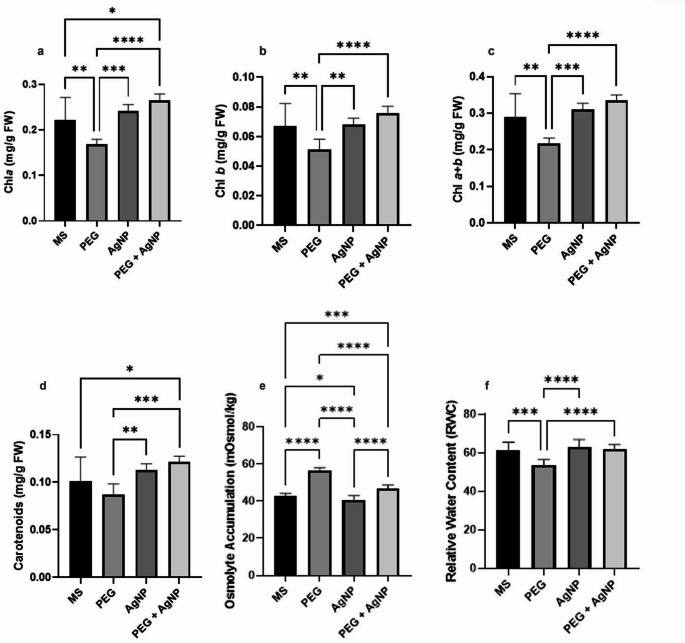
Physiological analysis of the plants under tested conditions. (**a** Chl *a* content, **b** Chl *b* ; **c** Chl *a + b*; **d** Carotenoid contents; **e** Osmolyte accumulation; **f** RWC) Asterisks represent statistically significant variations (*p ≤ 0.0001, **p ≤ 0.001, **p ≤ 0.01, *p ≤ 0.05), whereas non significant results, which are not shown in the figures

**Fig. 10:**
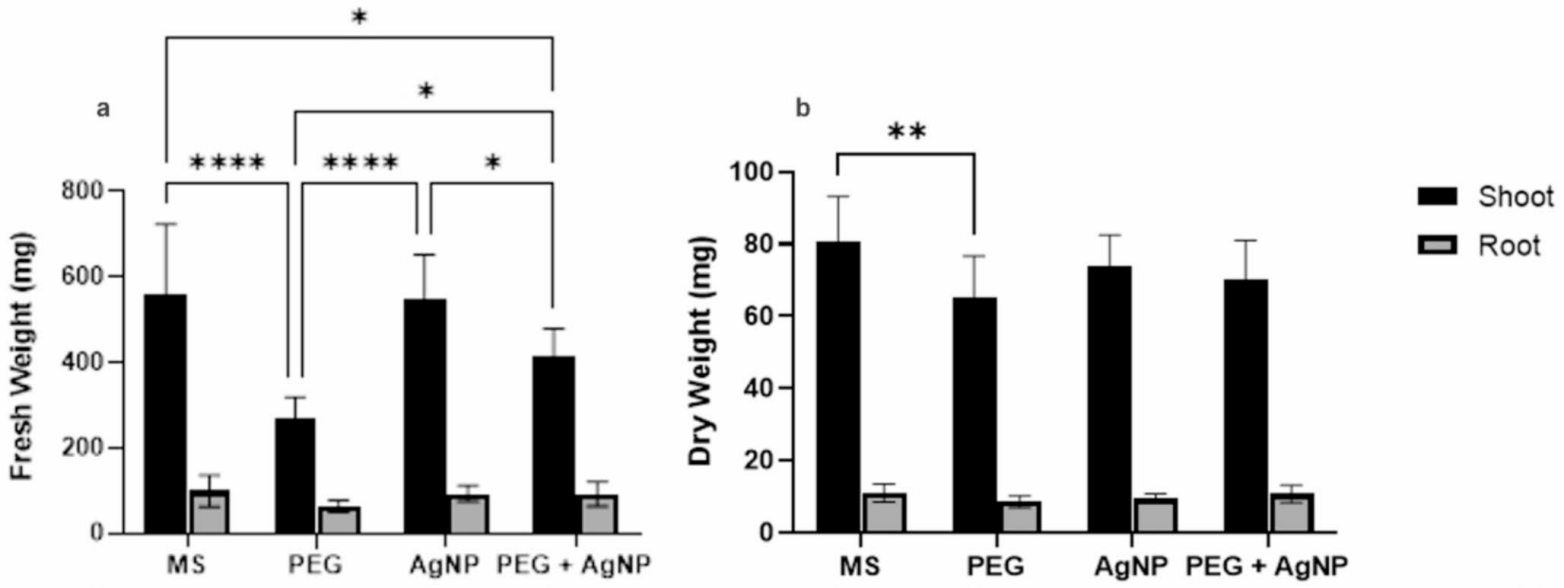
Biomass accumulation of the plants under different conditions. (**a** Fresh weights of shoots and roots of the plants under different conditions, **b** Dry weights of shoots and roots of the plants under different conditions.)

No significant differences were observed in DWs between MS, AgNP, and PEG + AgNP plants. The shoot DWs of PEG plants were lower than those of MS plants. Additionally, no significant differences in root FW or DW were detected across all conditions (Figs. 9 and 10). All physiological analysis results are summarized in Table 4, with mean values and standard errors.

**Table:** Table 4 Mean values and standard errors (SE) of physiological parameters measured under MS, PEG, AgNP, and PEG + AgNP treatments.

Physiological Analysis	MS	PEG	AgNP	PEG + AgNP
Relative water content (RWC)	61.28 ± 1.430	53.38 ± 1.043	62.93 ± 1.344	61.85 ± 0.7841
Osmolit Accumulation	42.89 ± 0.4231	56.78 ± 0.4648	40.33 ± 0.8819	46.89 ± 6.334
Chl *a*(mg/g FW)	0.2218 ± 0.0176	0.1684 ± 0.0042	0.2414 ± 0.005	0.2643 ± 0.0055
Chl *b*(mg/g FW)	0.06708 ± 0.0054	0.05127 ± 0.0023	0.06829 ± 0.0014	0.07599 ± 0.0015
Chl *a + b* (mg/g FW)	0.2888 ± 0.023	0.2168 ± 0.0059	0.3097 ± 0.0063	0.3350 ± 0.0062
Carotenoids (mg/g FW)	0.1012 ± 0.0089	0.0868 ± 0.0040	0.1126 ± 0.0024	0.1216 ± 0.1216
Fresh Shoot weight (mg)	557.2 ± 67.45	269.8 ± 20.03	544.7 ± 43.76	412.8 ± 27.1
Fresh Root weight (mg)	99.17 ± 15.01	63.17 ± 5.677	93.17 ± 7.867	91.50 ± 11.71
Dry Shoot weight (mg)	80.83 ± 5.049	65.00 ± 4.782	73.67 ± 3.639	70.00 ± 4.517
Dry Root weight (mg)	11.00 ± 1.033	8.500 ± 0.6708	9.333 ± 0.5578	10.67 ± 0.9888

### Effects of AgNPs on gene expression under drought stress

The expression of the *CAT* gene increased across all PEG, AgNP, and PEG + AgNP compared to the MS. The increases observed in PEG and PEG + AgNP were statistically significant, while the increase in the AgNP condition was not significant.

For the *POD* gene, the highest expression level was observed in the AgNP condition. This increase was statistically significant compared to both MS and PEG + AgNP conditions. In the PEG + AgNP condition, a lower level of gene expression was detected compared to MS, although the difference was not statistically significant.

The expression of the *Cu/ZnSOD* gene exhibited higher levels under PEG, AgNP, and PEG + AgNP conditions compared to MS; however, these increases were not statistically significant. For the *MnSOD* gene, which encodes proteins involved in an alternative antioxidant defense pathway, the highest expression level was observed under the AgNP condition. Significant increases in gene expression were also detected under the AgNP and PEG + AgNP conditions compared to MS, whereas the increase under the PEG condition was not statistically significant.

Regarding *MPK17* gene expression, a non-significant decrease was observed in the PEG condition compared to MS. However, increases were noted in the AgNP and PEG + AgNP conditions, with the AgNP condition showing a statistically increase in gene expression compared to all other conditions.

The analysis of *IDI-1* gene expression revealed that the AgNP condition exhibited the highest gene expression level. This increase was statistically significant compared to MS and PEG + AgNP conditions. While PEG and PEG + AgNP conditions also showed higher expression profiles compared to MS, these increases were not statistically significant.

**Fig. 11:**
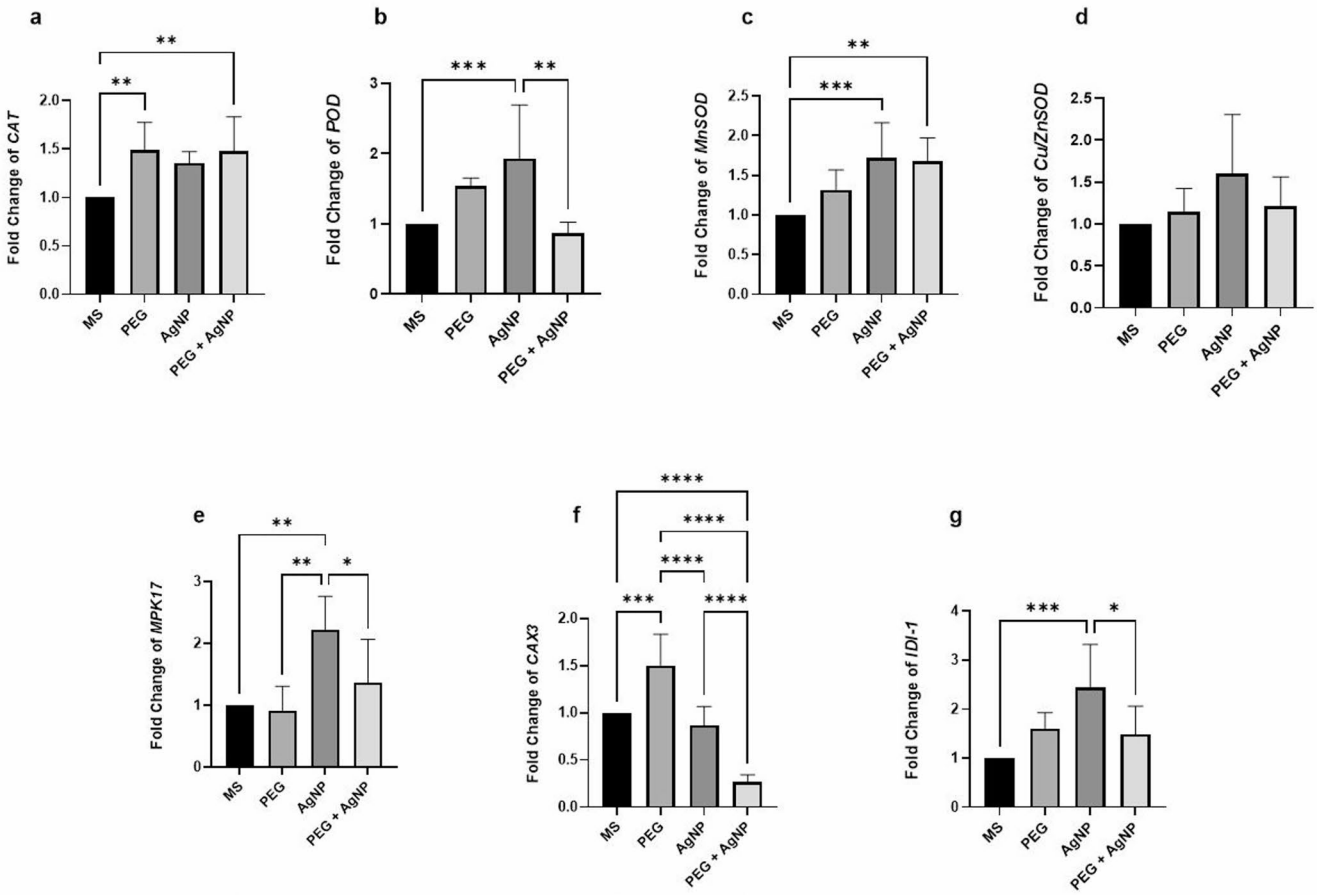
Statistically significant results of the different relative gene expression changes refer to *UBQ7* of the plant samples. (**a** *CAT*, **b** *POD*, **c** *MnSOD*, **d** *MPK17*, **e** *CAX3*, **f** *IDI-1)* Asterisks represent statistically significant variations (*p ≤ 0.0001, **p ≤ 0.001, **p ≤ 0.01, *p ≤ 0.05), whereas non significant results, which are not shown in the figures

The *CAX3* gene expression was the lowest in the PEG + AgNP condition, which was significantly reduced compared to all other conditions. No significant changes were detected between the AgNP and MS conditions. In contrast, the PEG condition exhibited a significant increase in gene expression compared to all other conditions Fig. 11 shows the statistically significant results of the gene expression analysis.

## Discussion

There are examples using plant waste sources in green synthesis, such as the synthesis of zinc oxide nanoparticles from onion, grape, lemon and orange peel wastes (Okpara et al. 2020; Modi et al. 2022), the synthesis of silver and gold nanoparticles from peel of pomegranate (Ahmad et al. 2012). Pérez-Alvarez et al. (2021) used waste cotton fabrics in the green synthesis of AgNPs. The green synthesis of SnO_2_ nanoparticles was carried out through agricultural boll waste of cotton (Narasaiah et al. 2022). Govarthanan et al. (2016) performed the green synthesis of AgNPs using cotton seed oil cake, a by-product of the cotton farming industry, and in this study, cotton seed oil cake was used for the green synthesis of AgNPs in a similar manner. This study contributes to sustainability by using CSOC, a by-product of the cotton production industry, as the starting material. Additionally, the use of the green synthesis method in this study aims to emphasize the importance of green chemistry principles. In this study the synthesis of AgNPs the experiment was carried out with 5 mM AgNO_3_ and 10% CSOCE. The characterization of the synthesized AgNPs was provided by UV-VIS, FTIR, DLS, XRD and SEM-EDS analyses.

The average size of particles was 256.5 nm, ZP was − 28.7 mV and the PDI was 0.65. Nevertheless, it is important to acknowledge that the hydrodynamic diameter measured during the DLS analysis includes the hydration layer around the particle, and therefore, the dimensions obtained do not represent the actual physical dimensions of the nanoparticles but their behavior in solution. For a more comprehensive assessment of the particle sizes, it is crucial to confirm DLS results with techniques such as TEM or SEM, which provide details such as the particle’s surface morphology and estimated surface area (Bhattacharjee 2016). Similarly, the particle size of green synthesized AgNPs using *Azadirachta indica* was determined 249 nm by DLS analysis but when it is examined by field emission scanning electron microscopy (FESEM) the particle sizes were less than 50 nm. This difference was obtained because of the hydrodynamic diameter (Alharbi and Alsubhi 2022). The PDI, a unitless measure indicating the heterogeneity in the size distribution of particles in the sample, is a crucial factor in evaluating the homogeneity level of nanoparticles. Systems with a PDI value greater than 0.7 are considered to have a broad particle size distribution (Nasiriboroumand et al. 2018). The PDI value of the AgNPs was 0.65, indicating that sample has moderate heterogeneity. Nguyen et al. (2023) green synthesized AgNPs, using *Callisia fragrans* extract. They reported PDI was 0.523 and they highlighted that under PDI values bigger than 0.7 are indicating the sample have broad in particle distribution. This assertion confirms that the PDI value obtained in our results indicates the formation of particles is moderate heterogeneity.

The zeta potential, a critical parameter that evaluates nanoparticles’ electrostatic interactions and colloidal stability depending on the surface charge (Shaw 1992). ZP between − 10 and + 10 mV indicates nanoparticles are neutral, values outside the range of + 30 and − 30 mV are considered an indicator of strong cationic or anionic effects (Clogston and Patri 2011). The ZP of the synthesized AgNPs, 28.7 mV, indicates particles are anionic in structure and have high colloidal stability due to their strong electrostatic repulsion forces. This high colloidal stability suggests that the AgNPs may have significant advantages in their use in biological media, highlighting their potential applications in this field. *C. lanceolatus*, *D. parviflorum*, *R. tomentosa*, *S. campanulatum* extracts have been used to synthesized AgNPs for investigating their antibacterial activity. The zeta potentials of the samples were differed between − 22.25 and − 28.44, similar to AgNPs synthesized in this study (Paosen et al. 2017).

SEM analysis indicated AgNPs were sizes of 50–100 nm and the spherical. The spherical structure of the AgNPs in the SEM images is consistent with the single SPR band EDS absorption peak at 3 keV specific to silver nanocrystals was observed. Similarly, Govarthanan et al. (2016) green synthesized AgNPs from CSOCE and determined that the AgNPs they synthesized were spherical and had variable sizes between 10 and 90 nm and Ag exhibited its own unique peak at 3 keV on EDS. In Vanti et al. (2018), AgNPs varying between 20 and 100 nm according to SEM *Gossypium hirsutum* shoot extract in the green synthesis of AgNPs these findings align with the results we obtained.

XRD peaks correspond to the reflections of fcc structure of AgNPs (Anis et al. 2023). Furthermore, there were undetermined peaks originating from the presence of biologically derived substances in the structure some of these peaks originate from AgCl formation (Anis et al. 2023l et al. 2024). The XRD spectrum of the study is consistent with the XRD results of nanoparticles obtained via green synthesis, as reported in the literature (Mirsadeghi et al. 2020).

After the green synthesis reaction, the color changed from pale yellow to dark brown, and the formation of the SPR band in the UV-VIS spectrum provides evidence that the phytochemicals in CSOC reduce Ag⁺ ions in the AgNO₃ solution to Ag⁰ through a redox reaction, thus confirming the formation of AgNPs. The sharp narrow range peak was observed at 430–450 nm in the UV-VIS spectrum indicating that AgNPs with small sizes and low aggregate formation were synthesized. Bhakya et al. (2016) investigated the antioxidant and antimicrobial activity of AgNPs synthesized from *Helicteres isora* root extract, synthesized AgNPs have been observed SPR band around 450 nm.

The peaks observed in FTIR wavelength of 3425 cm⁻¹ represent -OH groups in the structure. The peaks on 2922 cm⁻¹ and 2851 cm⁻¹ were attributed to the symmetric and asymmetric -CH and -CH₂ vibrations in the structure. The peak at 1613 cm⁻¹ indicates the presence of -C = O groups. 1464 cm⁻¹, 1386 cm⁻¹ and 1070 cm⁻¹ represents -CH₂ symmetric vibrations, C-H bonds and C-O groups originating from phytochemicals, respectively (Portella et al. 2016). AgNPs obtained by the green synthesis using *Gossypium hirsutum* leaves extract demonstrated the presence of FTIR bands at approximately 3422 cm⁻¹, 2922 cm⁻¹, 1629 cm⁻¹, 1381 cm⁻¹ and 651 cm⁻¹ wavelengths (Kanipandian and Thirumurugan 2014). From these spectral values, the peak at 2922 cm⁻¹ was attributed to the presence of asymmetric methylene C-H groups. Similarly, the FTIR spectrum of AgNPs synthesized from CSOCE by Govarthanan et al. (2016) showed peaks at approximately 3299 cm⁻¹, 2918 cm⁻¹, 1653 cm⁻¹ and 1051 cm⁻¹ wavelengths, the peak observed at 3299 cm⁻¹ was attributed to the presence of O-H groups of alcohols and phenols, and the peak at 1653 cm⁻¹ to C = C chemical groups of metabolites found. FTIR spectra of the synthesized AgNPs in our study, revealed highly consistent results with those of these literatures also using *Gossypium hirsutum* derived extracts, significantly contributing to the confirmation of the chemical structure. HPLC analyses determined secondary metabolites with the highest content were determined to be rhein, chrysophanol, and fission. FTIR analysis showed that -C = O, -OH, -CH and -CH₂ vibrations detected in the AgNP structure primarily originated from these three components; additionally, phytochemicals and plant cellulose structures found in the CSOCE structure.

Even though AgNPs are among the most frequently studied nanoparticles, they are typically investigated for their antimicrobial, antifungal and anticancer properties (Bibi et al. 2022; Khatami et al. 2018; Sánchez-Navarro et al. 2018). In the field of agriculture, the applicability of AgNPs to enhance the tolerance of plants under stress conditions is a relatively new area of research. AgNPs provide very effective results in increasing the germination rate and salt tolerance of *Cuminum cyminum* L. seeds under salinity stress (Abasi et al. 2022). AgNPs, the resistance of tomato seeds under NaCl stress increased, which increased the germination rate as well as the FW and DW of the seedling and root length. Sharon et al. (2010) showed that silver in soil and hydroponic systems repels microorganisms, promotes plant growth, and protects the plant from various diseases. In plants, AgNPs have been shown to effectively alleviate salt stress (Almutairi 2016; Abou-Zeid and Ismail 2018). AgNPs enhanced water balance, shoot length, and biomass in lentils under drought conditions and increased germination rates (Hojjat 2016). The effects of AgNPs on drought stress, which is the most impactful abiotic stress condition affecting plant growth and development, are quite limited (Farooq et al. 2009; Alfosea-Simón et al. 2025). Our primary objective with this study is to contribute to literature in this regard. *Gossypium hirsutum* L. plants germinated in in vitro conditions were then cultured for 72 h in PEG, AgNP and PEG + AgNP media representing the experimental conditions. Studies utilizing in vitro tissue culture techniques with cotton plants to investigate stress conditions are quite limited, and this study introduces novel contributions to the literature in this manner. Morphological, physiological, and gene expression levels were examined to assess the effects of the tested conditions.

A significant difference in shoot length was observed between plants in the PEG and PEG + AgNP conditions, while no significant difference was detected between the control and PEG + AgNP plants. AgNP treatment under drought stress mitigated the adverse effects of drought on shoot length. Furthermore, shoot lengths in the AgNP and PEG + AgNP conditions were comparable to the control, indicating that AgNP application did not have a negative impact on shoot growth.

Minimum values for LSA were recorded in plants subjected to PEG treatment, with this reduction being significantly different from all other conditions. However, the application of PEG + AgNP resulted in a leaf surface area closer to that of the control plants, demonstrating that PEG + AgNP treatment alleviated the drought-induced suppressive effects on leaf surface area. The AgNPs synthesized using *Moringa oleifera* extract have been found to improve the morphological and physiological characteristics of *Triticum aestivum* L. (Sabir et al. 2018). In this study, even though the MS and AgNP conditions have not significant differences in the terms of morphological analysis, there was significant differences between the PEG and PEG + AgNP, which indicates that AgNP can be used for enhancing morphological characteristics under drought stress.

RWC decreases under drought conditions and maintaining high RWC is considered an important indicator for drought tolerance (Abdelmoghny et al. 2020). Decreased RWC values were not observed only in PEG treatment due to drought. The fact that PEG + AgNP showed RWC values more similar to the MS control than to PEG alone provides evidence for the mitigating effect of AgNP on drought stress, specifically in preventing RWC reduction in plants under drought conditions. AgNP application to pearl millet seeds alleviated the negative effects of salinity stress RWC decreased under salinity stress, could be preserved by applying AgNP (Khan et al. 2020).

Plants accumulate organic osmolytes in response to environmental stresses that cause cellular dehydration. The increase in cellular osmolarity from solute accumulation helps water entry into cells or reduces its outflow, thus maintaining the turgor needed for cell growth (Hare et al. 1998). The significant difference between PEG and PEG + AgNP indicated that AgNP application helped to reduce osmolyte accumulation, which is an effect of drought stress, and exhibited osmolyte accumulation closer to control plants. Drought stress inhibits growth in plants, reduces plant productivity, causes stomata to close, and thus reduces photosynthesis (Németh et al. 2002).

Chlorophyll plays a direct role in carbohydrate metabolism during the photosynthesis process. Chlorophyll content decreases under drought stress, which disrupts the main components of photosynthesis and ultimately affects the growth, yield and biochemical processes of the plant and damages the plant by causing oxidative stress (Rao et al. 2012). Plants in the PEG condition had the minimum values ​​of Chl *a*, Chl *b* and Chl *a + b* contents compared with other conditions as a result of drought stress. The significant difference between PEG and PEG + AgNP indicates that AgNP application eliminated the negative effects of drought stress related to chlorophyll content under in vitro drought stress. AgNP application had results close to the control is evidence regarding AgNP application did not have a negative effect on the chlorophyll content. Similar to these findings, green synthesized AgNPs using *Moringa oleifera* extract alleviates the negative effects of heat stress in wheat. It has been determined that RWC, chl *a* and chl *b* contents increased in 50 mg/L AgNP application simultaneously with heat stress (Iqbal et al. 2019).

Rawson and Turner (1982) reported that water stress primarily reduces overall biomass production in plant leaves. The difference in fresh weight (FW) of shoots between the PEG + AgNP and PEG treatments supports the conclusion that AgNP application under in vitro drought stress alleviates the reduction in biomass caused by drought. Consistent with our findings, the application of AgNPs to soil contaminated with heavy metals led to increased biomass and chlorophyll pigment content in Chinese cabbage (Li and Huang 2014). Similarly, AgNP treatment has been shown to enhance shoot and root development and increase overall biomass in rice plants (Gupta et al. 2018). Moreover, AgNP application improved growth parameters, including biomass accumulation, in *Trigonella foenum-graecum* (Sadak 2019).

All physiological and morphological data were evaluated and as explained in detail above, no significant difference was detected between the control and AgNP conditions in most of the analyses performed. This could be related to the concentration of AgNPs applied, and aspect holds potential as a new research topic. Changes observed in the PEG condition were evaluated within the scope of literature, morphological and physiological changes developed as a result of in vitro drought stress. Differences between the PEG and PEG + AgNP treatments supported the main objective of the study: AgNP application to plants under drought stress mitigated the negative changes that occurred in the plant as a result of drought stress. Results showing that AgNP application yielded values closer to the MS condition than to PEG suggest that AgNPs did not negatively affect the plants, and this observation is in line with findings from previous studies. Understanding the mechanism of nanoparticle effects on plants and identifying potential positive and negative impacts requires increased research in the field. To diversify research in this area, more studies are needed to determine the efficacy, lifespan, toxicity, and antioxidant parameters of AgNPs in photosynthetic systems.

Gene expression changes in plants under MS, PEG, AgNP and PEG + AgNP conditions were examined for certain genes. Ct values ​​obtained from qPCR results for *CAT*, *POD*, *Cu/ZnSOD*, *MnSOD*, *MPK17*, *IDI-1* and *CAX3* genes were evaluated.

*CAT* genes increased and the expression of *Cu/ZnSOD* genes decreased depending on the AgNP application in their studies where they investigated the stimulatory effect of biosynthesized AgNPs on *Oryza sativa* L. plants (Gupta et al. 2018). *CAT* gene expressions increased under AgNP and PEG + AgNP conditions, similarly. Hu et al. (2021) examined the effects of abscisic acid (ABA) and melatonin (MT) application under drought stress of *Gossypium hirsutum* L. Expressions of *POD*, *Cu/ZnSOD* and *MnSOD* genes increased under the effect of drought stress, while the expression of *CAT* gene decreased. It was reported that all antioxidant gene activities increased as a result of the applications helping the antioxidant system. In this study, antioxidant gene activities that increased in a similar way were accepted as evidence that there was an increase in gene expression as a result of the applications that would help the defense of the antioxidant system. The decrease in *POD* gene expression only in the PEG + AgNP condition can already be explained by the high POD enzyme concentration in the plant or the use of non-enzymatic defense pathways in addition to the enzymatic antioxidant system. Understanding the effects of AgNPs on plants oxidative system is a complex and encompasses multiple dimensions, requiring comprehensive and detailed exploration (Noori et al. 2024). To achieve a more comprehensive understanding of this issue, analyzing antioxidant enzyme activities may provide additional data, and could be a research area for future studies.

Mitogen-activated protein kinase (MAPK) signaling pathway is a complex and highly conserved in eukaryotes. The pathway has important roles in the response to various biotic and abiotic stresses (Sinha et al. 2011). As an important signal transducer, MAPK pathway plays an important role in drought stress via ABA-dependent signaling pathway and regulating reactive oxygen species (ROS) production (Ding et al. 2013). Zhang et al. (2014) identified *GhMPK17*, a MAPK gene in cotton. They observed that the *GhMPK17* gene was predominantly expressed in cotyledons and anthers, and its expression was also triggered by salt stress, osmotic stress, and ABA. Overexpression of the gene in Arabidopsis increased the tolerance of the plant to salt and osmotic stress, indicates *GhMPK17* plays a role in multiple abiotic tolerance of cotton. In this study, no significant gene expression change was determined in PEG and PEG + AgNP conditions compared to MS. The gene expression level in the AgNP condition was statistically significant higher than in MS. Rodriguez-Uribe et al. (2014) performed microarray analysis to identify drought-sensitive genes in *Gossypium hirsutum* L. The study reported 88 genes suppressed by drought. Two of these suppressed genes were found to be *IDI-1* and *CAX3* genes, whose expression analysis was also examined in study. Calcium (Ca^2+^) as a secondary messenger plays an important role in signal transduction in various abiotic stresses. During stress, the amount of [Ca^2+]^cyt increases temporarily and this is known as the calcium signature. *CAX*s (Ca^2+/^H^+^ exchangers) play a critical role in this process. Xu et al. (2013) isolated and characterized a putative Ca^2+^/H^+^ exchanger *GhCAX3* gene from *Gossypium hirsutum* for the first time. They stated that overexpression of *GhCAX3* could increase the activities of some cold and ABA-sensitive marker genes. In a study investigating the effects of melatonin application against salinity stress in *Gossypium hirsutum*, it was reported that the expression of the *CAX3* gene increased in some applications and decreased in others in a manner not directly proportional to the application dose (Ren et al. 2022). In this study, *CAX3* gene expression increased significantly in PEG application compared to MS, while it decreased significantly in PEG + AgNP application. The gene expression profile of AgNP application did not have a significant difference with MS application. Isopentenyl diphosphate isomerase (IDI) catalyzes the interconversion of isopentenyl diphosphate (IPP) and dimethylallyl diphosphate (DMAPP) and plays a role in the synthesis of isoprenoids (terpenoids), the most structurally and functionally diverse group of plant metabolites (Buckingham 1995). *IDI-1*, involves protonation and subsequent deprotonation in isoprenoid pathways in (Wu et al. 2005). In plants, this is a reaction that occurs in plastids and has an important role in plant metabolism. DMAPP forms precursor molecules of sterols, carotenoids, and various plant hormones. It is known that under drought stress the amount of enzymes involved in isoprenoid biosynthesis decreases in *Zea mays* L. (Ren et al. 2022). According to the findings obtained in this study, no significant difference was detected in the expression levels of the *IDI-1* gene in PEG and PEG + AgNP applications compared to MS. However, gene expression in the AgNP condition increased significantly compared to MS. This increase may be an indication that AgNP application has a stimulating effect on isoprenoid metabolism. Our findings regarding the expression of the *CAX3* and *IDI-1* genes differ from those in literature and present this observation for the first time. The fact that *CAX3* and *IDI-1* genes are not suppressed under drought stress, unlike the examples in the literature, may be due to the intensity and duration of drought application. Studies investigating the expression changes of these genes under AgNP applications have not been found in the literature. In addition, since gene expression analysis may not be directly related to the amount of enzyme and protein, further studies are needed to comment on this issue.

This study is first to demonstrate the mitigating effects of AgNPs synthesized from CSOCE, derived from cotton plant harvest waste, on in vitro drought stress in cotton plants. Due to its innovative approaches in many aspects, this study can serve as a model for future research. The methodology and findings of this study open a wide range of possibilities for future research and more detailed investigations. This study, as previously reported by Govarthanan et al. (2016), utilized CSOCE for the green synthesis of AgNPs, and the formation of AgNPs was demonstrated through various characterization methods. Future studies could explore the synthesis of other metallic nanoparticles using CSOCE. The synthesized nanoparticles were applied in 50 mg/L insights of studies have done before, the potential effects of varying concentrations of AgNPs on cotton plants under drought stress, including whether increasing doses may result in cell viability and toxic outcomes, are among the topics that could be investigated in future studies. Even though it was not observed in this study, there are reports about the toxic effects of silver nanoparticles on plants (Oukarroum et al. 2013; Yan and Chen 2019). Investigating the use of different application methods is a further crucial research topic. As is known, the uptake and transport of nanoparticles by plants can occur through various pathways (Khan et al. 2022; Wang et al. 2023). Determining the most suitable nanoparticle application method under in vivo conditions could be one of the key research areas for advancing the method. In addition to all these studies, by modeling a field under drought conditions and conducting in vivo tests. The potential usage of green synthesized AgNPs as nano fertilizers or stress reducing agents constitutes a promising research area.

## Conclusion

In this study, AgNPs were synthesized via green synthesis using CSOC, a by-product of the cotton industry. Morphological, physiological, and gene expression analyses revealed significant findings, indicating that the synthesized and applied AgNPs effectively mitigate the adverse effects of drought stress in in vitro grown *Gossypium hirsutum* plants. The results demonstrated that AgNP application under drought stress exhibited a notable phytostimulatory effect, reducing the morphological and physiological damage caused by drought stress.

This study highlights sustainability by adopting an environmentally friendly approach, utilizing cottonseed oil cake as a raw material for nanoparticle synthesis. Research on the effects of green synthesized AgNPs on drought stress is still limited, and this study provides valuable insights to the existing literature. The effects of green-synthesized AgNPs using CSOC extract on drought stress were investigated for the first time, and the findings demonstrated that they mitigate the suppressive effects of drought stress on plants. Further research is essential to explore the potential of AgNPs in mitigating the negative impacts of different abiotic stress conditions on plants. The findings of this study also support the potential role of AgNPs in alleviating drought stress and are anticipated to inspire future investigations in this area.

## Electronic supplementary material

Below is the link to the electronic supplementary material.

Supplementary Material 1

## Data Availability

The data that support the findings of this study are available from the corresponding author upon reasonable request. All methods were performed in accordance with relevant guidelines and regulations.
