# Supplementary material for: Green synthesized silver nanoparticles enhance drought tolerance in cotton plants cultured in vitro
Source: Physiol Mol Biol Plants. 2025 Jun 28;31(6):959–78. doi: 10.1007/s12298-025-01616-z (PMC12314283; doi:10.1007/s12298-025-01616-z)
Supplement: Supplementary file 1 — Supplementary Material 1 [file 12298_2025_1616_MOESM1_ESM.pdf]

agparticle

Author: support

Creation: 10/24/2023 12:07:14 PM

Sample Name: 241123_3

Added Spectra

10/24/2023 12:07:14 PM

kV: 30	Mag: 60000	Takeoff: 32	Live Time(s): 30	Amp Time(µs): 7.68	Resolution:(eV) 128.6

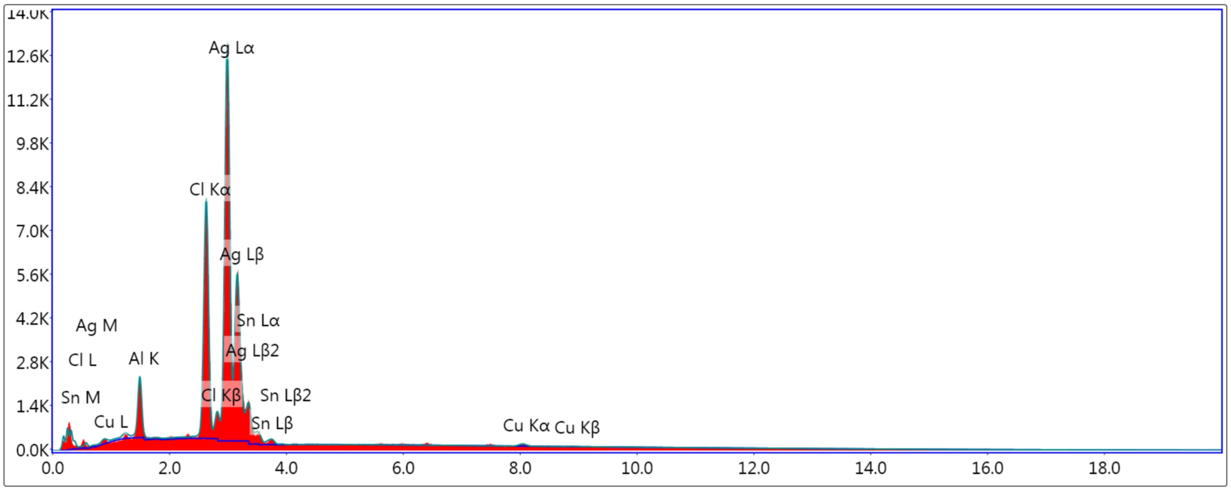

Lsec: 30.0 0 Cnts 0.000 keV Det: Octane Super Det Reso

eZAF Smart Quant Results

Element	Weight %	Atomic %	Net Int.	Error %	Kratio	Z	R	A	F	KABFactor
AlK	7.51	18.85	975.26	8.94	0.02670448	1.180609		0.2989451	1.007758	
ClK	17.85	34.11	4602.68	4.17	0.1425069	1.140019		0.6830934	1.025028	
AgL	71.67	45.01	8364.13	2.28	0.5882235	0.9510478		0.8624015	1.000613	
SnL	2.29	1.3	148.16	19.75	0.01235637	0.9161534		0.5921378	0.9965849	
CuK	0.68	0.73	72.92	21.29	0.006252384	1.031743		0.8891545	1.001249	
